# Sensitivity to thyroid hormone indices are associated with papillary thyroid carcinoma in Chinese patients with thyroid nodules

**DOI:** 10.1186/s12902-023-01381-8

**Published:** 2023-06-01

**Authors:** Jie Sun, Jie Liu, Ting-ting Wu, Zhi-yuan Gu, Xiao-wen Zhang

**Affiliations:** 1 Department of Endocrinology, Endocrine and Metabolic Disease Medical Center, Nanjing Drum Tower Hospital, Affiliated Hospital of Medical School, Nanjing University, Nanjing, China; 2 Branch of National Clinical Research Centre for Metabolic Diseases, Nanjing, China; 3 Department of Endocrinology, Endocrine and Metabolic Disease Medical Center, Nanjing Drum Tower Hospital, Chinese Academy of Medical Sciences & Peking Union Medical College, Nanjing, China

**Keywords:** Central thyroid hormone sensitivity, Peripheral thyroid hormone sensitivity, Papillary thyroid carcinoma, TNM staging, Prevention

## Abstract

**Background:**

The association between thyroid hormone sensitivity and thyroid cancer is unknown, and we aimed to investigate the association between sensitivity to thyroid hormone indices and papillary thyroid carcinoma (PTC) in Chinese patients with thyroid nodules (TNs).

**Methods:**

A total of 1,998 patients undergoing thyroid surgery due to TNs from Nanjing Drum Tower Hospital were included in this study. We evaluated central sensitivity to thyroid hormones, such as thyroid stimulating hormone index (TSHI), TSH T4 resistance index (TT4RI), thyroid feedback quantile-based index (TFQI), and parametric thyroid feedback quantile-based Index (PTFQI). Peripheral sensitivity to thyroid hormone was evaluated by FT3 to FT4 ratio. Multivariate logistic regression analysis was performed to evaluate the association between sensitivity to thyroid hormone indices and PTC risk.

**Results:**

The results showed that central indices of thyroid hormone sensitivity, including TSHI, TT4RI, TFQI, and PTFQI, were positively associated with PTC risk. For each SD increase in TSHI, TT4RI, TFQI, and PTFQI, the odds ratios (OR, 95% CI) of PTC were 1.31 (1.18–1.46), 1.01 (1.01–1.02), 1.94 (1.45–2.60), and 1.82 (1.41–2.34), respectively. On the other hand, the association between peripheral sensitivity to thyroid hormone and PTC was significantly negative. For each SD increase in FT3/FT4 ratio, the OR (95% CI) of PTC was 0.18 (0.03–0.96), and a negative correlation was found between FT3/FT4 ratio and TNM staging of PTC.

**Conclusions:**

Sensitivity to thyroid hormone indices could be used as new indicators for predicting PTC in Chinese patients with TNs. Future researches are still needed to confirm our findings.

**Supplementary Information:**

The online version contains supplementary material available at 10.1186/s12902-023-01381-8.

## Background

The incidence of thyroid cancer has been progressively increasing over the past decades, becoming the most pervasive endocrine malignancy worldwide [1, 2]. In China, the incidence rate of thyroid cancer surged from 2.40 per 100,000 people in 2003 to 13.75 per 100,000 people in 2012, showing a 4.73 fold increase, with an average annual increase of 20% [3]. Papillary thyroid carcinoma (PTC) is the most common subtype and accounts for over 80% of cases [4]. Although it is well-known that female gender, aging, ionizing radiation exposure, and family history have been established as risk factors [5–8], the causal factors of PTC remain unknown.

Thyroid-stimulating hormone (TSH) is secreted by the pituitary gland to support the growth and biological function of thyroid cells. Previous researches have investigated the association between TSH and thyroid cancer, but their results were inconsistent [9]. Both hyperthyroidism and hypothyroidism have been linked to thyroid cancer [10–12]. Besides, even within the normal range, changes in thyroid function may be associated with the risk of thyroid cancer [9]. These conflicting findings indicate that the underlying mechanism of the relationship between thyroid parameters and thyroid cancer involves complex pathophysiological processes. Recently it is worth noting that common people might exhibit moderately acquired resistance to thyroid hormones. Changes in thyroid hormones sensitivity, present in both thyroid hormone excess and deficiency, are associated with metabolic diseases, cardiovascular disease, kidney disorders, and all-cause mortality [13–17]. To our knowledge, no research has reported the relationship between sensitivity to thyroid hormones with PTC till now. Thus, we conducted a case–control study in order to examine the association between sensitivity to thyroid hormone indices and the PTC risk in 1,998 patients aged 20 years and older who underwent thyroid surgery due to thyroid nodules (TNs) from Jiangsu province of eastern China.

## Methods

### Study population

**Fig. 1:**
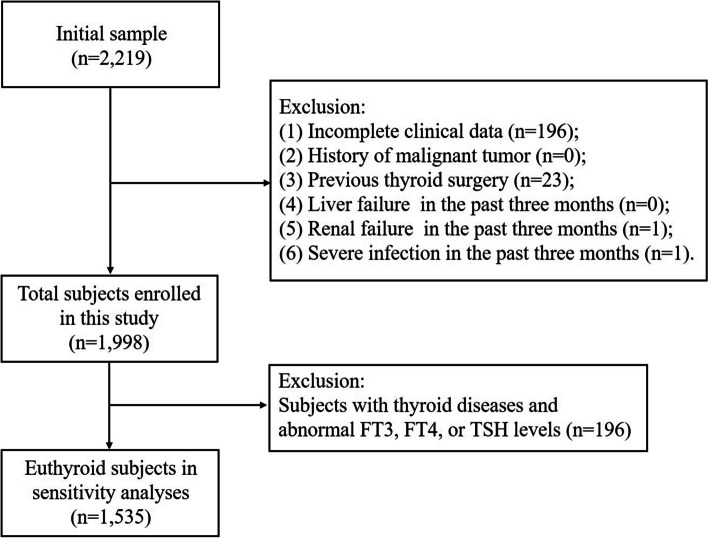
Flowchart of the inclusion and exclusion of participants

The participants consisted of 2,219 patients aged 20 years and above who underwent thyroid surgery due to TNs in Thyroid Surgery Department of Nanjing Drum Tower Hospital (Nanjing, China) from January 2009 to December 2017. The exclusion criteria were as follows: (1) incomplete clinical data (*n* = 196); (2) history of malignant tumor (*n* = 0); (3) previous thyroid surgery (*n* = 23); (4) liver failure, renal failure, or severe infection in the past three months (*n* = 2). This resulted in the inclusion of 1,998 individuals (men, 521; women, 1,477) in the final retrospective cross-sectional study. In addition, we excluded subjects with thyroid diseases and those with abnormal free triiodothyronine (FT3), free thyroxine (FT4), or TSH levels for sensitivity analyses. The reference ranges of FT3, FT4, and TSH were 3.1–6.8 pmol/L, 12–22 pmol/L, and 0.27–4.20 mIU/L, respectively. After exclusion, 1,535 individuals were included in sensitivity analyses (Fig. 1). Before undergoing thyroid surgery, all recruited patients were given informed consent by trained staff. Demographic data (e.g., age and sex), lifestyle factors, medical history, family history, and history of ionizing radiation exposure were collected using a structured questionnaire, followed by a physical examination and blood sampling. Clinical information, such as tumor histology and TNM staging [18], was obtained from the patients’ medical records after the surgery. According to the pathologic results, 1,457 patients with PTC and 541 patients with benign TNs were confirmed.

### Body measurements

Weight and height were measured to calculate body mass index (BMI) by medical staff. Blood pressure was measured twice on the right arm after 15 min sitting using standard instruments, and the average of both measurements was taken as the patient's blood pressure.

### Laboratory examinations

Biochemical markers were measured in the clinical laboratory of Nanjing Drum Tower Hospital, which carried out laboratory tests according to ISO15189 international quality standard. Approximate 5-ml of venous blood was drawn from each subject in the morning, after a 12-h overnight fast. After collection, the blood sample was immediately sent to the clinical laboratory for centrifugation and testing through the transmission system. The remaining serum was stored at 2–8 °C for two weeks before discarding, unless there was any question about the test results. Fasting plasma glucose (FPG), total cholesterol (TC), triglyceride (TG), high-density lipoprotein cholesterol (HDL-C), and low-density lipoprotein cholesterol (LDL-C) concentrations were measured on enzymatic auto-analyzer (Kyowa Medex Co., Ltd. Tokyo, Japan) according to the manufacturer’s instructions. Serum levels of FT3, FT4, TSH, thyroglobulin antibody (TgAb), and thyroid peroxidase antibody (TPOAb) were measured by electrochemiluminescence (Roche Diagnostics, Basel, Switzerland) following the standard methods with strict quality control. The intraassay coefficients of variation (CV) for serum FT3, FT4, TSH, TgAb, and TPOAb were 15–22%, 14–29%, 15–86%, 24–56%, and 22–63%, respectively. The inter-assay CV values were 17–28%, 27–66%, 18–87%, 21–69%, and 52–82%, respectively.

### Indices of thyroid hormone sensitivity

FT3/FT4 ratio was used to evaluate the peripheral thyroid hormone sensitivity. Central indices of thyroid hormone sensitivity included TSH index (TSHI), TSH T4 resistance index (TT4RI), Thyroid Feedback Quantile-based Index (TFQI), and Parametric Thyroid Feedback Quantile-based Index (PTFQI). TSHI = ln TSH (mIU/L) + 0.1345 * FT4 (pmol/L) [19]. TT4RI = FT4 (pmol/L) * TSH (mIU/L) [20]. TFQI and PTFQI were calculated using the algorithm developed by Laclaustra et al. [13]. Using the population empirical cumulative distribution function (cdf) of FT4 and TSH, TFQI was determined by the formula of cdf FT4 − (1 − cdf TSH) in the current population. In addition, we calculated PTFQI, an estimated value of TFQI that available for reference population, by the formula of Φ((FT4 − μFT4)/σFT4) − (1 − Φ((ln TSH − μln TSH)/σln TSH)). For Chinese, we defined μFT4 = 17.0242, σFT4 = 2.05301, μln TSH = 0.6918, and σln TSH = 0.44546, which calculated from 8,332 subjects aged 20 years and above with normal thyroid function undergoing a regular physical examination at Nanjing Drum Tower Hospital during 2010 to 2016. To enhance the representativeness and reliability of the aforementioned four parameters (μFT4, σFT4, μln TSH, and σln TSH), the age and gender distribution of these 8,332 subjects was in according with that of the general population in China based on Chinese census data in 2010.

### Statistical analysis

Continuous variables were shown as median (interquartile ranges, IQRs), and the categorical ones were shown as number of events (percentages). We performed the Mann–Whitney U test for continuous variables and the χ^2^ test for categorical variables to analyze the differences in characteristics between PTC cases and the controls which were patients with benign TNs. Comparisons of the thyroid parameters of patients with stage I and stage II PTC were conducted using the Mann–Whitney U test. To investigate the association between per SD change of thyroid parameters and PTC, we analyzed multivariate logistic regression in two models: model 1 adjusted for age and gender; while model 2 adjusted for age, gender, BMI, systolic blood pressure (SBP), diastolic blood pressure (DBP), FPG, TC, TG, HDL-C, LDL-C, TgAb, TPOAb, history of ionizing radiation exposure, and family history of thyroid cancer. Comparisons of PTC risk across quartiles of thyroid parameters, where the first quartile was set as reference, were further carried. The odds ratios and 95% confidence intervals were given. The correlations between thyroid parameters and the TNM staging of PTC were determined by using spearman correlation analyses. All these statistical analyses were performed using R software (version 4.0.5, 2021–03-31). The significance level was set at *P* < 0.05 and *P* values were given for 2-sided tests.

## Results

### Characteristics of the study population

The characteristics of the study population were summarized in Table 1. There was no statistically significant difference between the patients with PTC and those with benign TNs in terms of sex, BMI, DBP, TC, TG, LDL-C, TPOAb, FT3, FT4, FT3/FT4, family history of thyroid cancer, use of levothyroxine, and use of anti-thyroid drugs. TgAb (*P* < 0.001), TSH (*P* < 0.001), TSHI (*P* < 0.001), TT4RI (*P* < 0.001), TFQI (*P* < 0.001), and PTFQI (*P* < 0.001) were significantly higher in the patients with PTC compared with those with benign TNs. However, SBP (*P* < 0.001), FPG (*P* < 0.001), and HDL-C (*P* = 0.004) were significantly lower in the patients with PTC compared with those with benign TNs. The patients with PTC (43 [range, 33–52] years old) were significantly younger than those with benign TNs (52 [range, 44–60] years old). Besides, the proportion of patients with history of exposure to ionizing radiation in PTC cases was higher than that in controls, and the difference was statistically significant (*P* = 0.011).

**Table 1:** Characteristics of patients with thyroid nodules

Characteristics	Total	Benign TNs	PTC	*P* value
No. of patients (%)	1,998 (100.0)	541 (27.1)	1,457 (72.9)	
Age (years)	46 (34, 55)	52 (44, 60)	43 (33, 52)	< 0.001
Male, n (%)	521 (26.1)	129 (23.8)	392 (26.9)	0.169
BMI (kg/m^2^)	23.57 (21.37, 25.78)	23.80 (21.49, 25.65)	23.51 (21.33, 25.78)	0.329
SBP (mmHg)	126 (116, 137)	128 (118, 139)	125 (115, 136)	< 0.001
DBP (mmHg)	77 (70, 84)	77 (70, 84)	77 (70, 84)	0.694
FPG (mmol/L)	4.46 (4.19, 4.82)	4.55 (4.28, 4.88)	4.44 (4.16, 4.79)	< 0.001
TC (mmol/L)	4.18 (3.65, 4.74)	4.21 (3.62, 4.79)	4.17 (3.66, 4.72)	0.663
TG (mmol/L)	1.07 (0.75, 1.56)	1.06 (0.76, 1.53)	1.07 (0.74, 1.58)	0.736
HDL-C (mmol/L)	1.16 (0.97, 1.39)	1.19 (0.99, 1.42)	1.15 (0.96, 1.38)	0.004
LDL-C (mmol/L)	2.41 (1.99, 2.89)	2.44 (1.99, 2.88)	2.40 (2.00, 2.89)	0.956
History of exposure to ionizing radiation, n (%)	46 (2.3)	5 (0.9)	41 (2.8)	0.011
Family history of thyroid cancer, n (%)	89 (4.5)	16 (3.0)	73 (5.0)	0.051
TgAb (IU/mL)	11.36 (10.00, 35.83)	10.60 (10.00, 16.25)	11.88 (10.00, 60.95)	< 0.001
TPOAb (IU/mL)	17.20 (11.60, 26.20)	16.90 (11.85, 23.30)	17.50 (11.42, 28.47)	0.060
FT3 (pmol/L)	4.94 (4.57, 5.35)	4.95 (4.58, 5.32)	4.93 (4.56, 5.35)	0.614
FT4 (pmol/L)	16.79 (15.20, 18.58)	16.80 (15.09, 18.72)	16.75 (15.20, 18.51)	0.873
TSH (mIU/L)	2.35 (1.56, 3.52)	1.95 (1.22, 3.13)	2.46 (1.69, 3.72)	< 0.001
TSHI	3.12 (2.68, 3.55)	2.93 (2.47, 3.38)	3.19 (2.77, 3.59)	< 0.001
TT4RI	39.14 (26.04, 59.37)	32.77 (20.70, 50.77)	41.25 (28.29, 62.21)	< 0.001
TFQI	0.01 (-0.24, 0.24)	-0.06 (-0.31, 0.15)	0.03 (-0.21, 0.27)	< 0.001
PTFQI	0.05 (-0.20, 0.34)	-0.02 (-0.29, 0.22)	0.09 (-0.15, 0.38)	< 0.001
FT3/FT4	0.30 (0.27, 0.33)	0.30 (0.26, 0.33)	0.30 (0.27, 0.33)	0.989
Use of levothyroxine, n (%)	67 (3.4)	21 (3.9)	46 (3.2)	0.510
Use of anti-thyroid drugs, n (%)	11 (0.6)	3 (0.6)	8 (0.5)	1.000

Data are expressed as median (interquartile ranges) or frequency (%)

*TNs* Thyroid nodules; *PTC* Papillary thyroid carcinoma; *BMI* Body mass index (weight/height2); *SBP* Systolic blood pressure; *DBP* Diastolic blood pressure; *FPG* Fasting plasma glucose; *TC* Total cholesterol; *TG* Triglyceride; *HDL-C* High-density lipoprotein cholesterol; *LDL-C* Low-density lipoprotein cholesterol; *TgAb* Thyroglobulin antibody; *TPOAb* Thyroid peroxidase antibody; *TSH* Thyroid-stimulating hormone; *FT3* Free triiodothyronine; *FT4* Free thyroxine; *TSHI* TSH index; *TT4RI* TSH T4 resistance index; *TFQI* Thyroid Feedback Quantile-based Index; *PTFQI* Parametric Thyroid Feedback Quantile-based Index

### Association of sensitivity to thyroid hormones with the risk of PTC

**Fig. 2:**
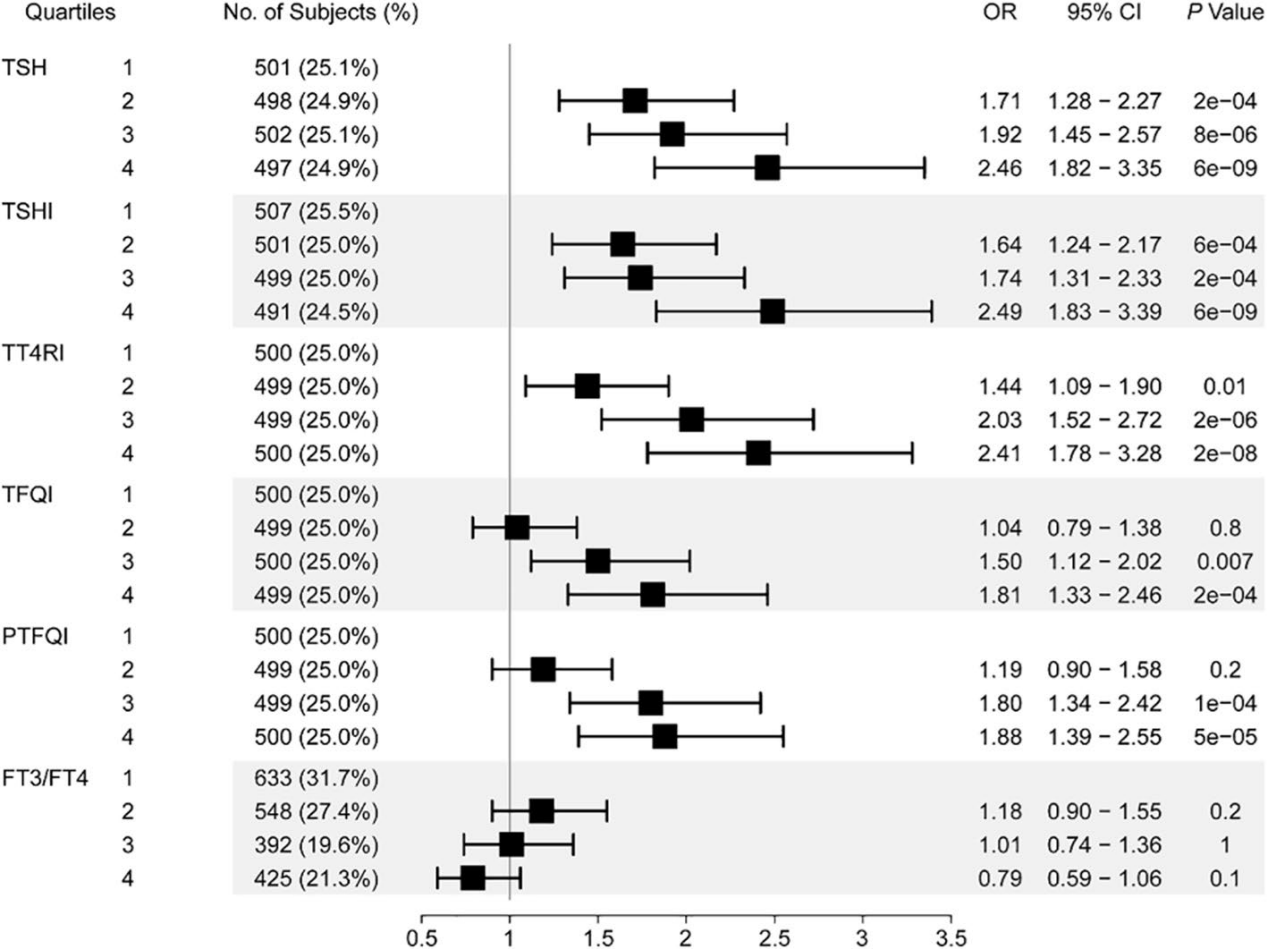
Association between quartiles of thyroid parameters and prevalence of papillary thyroid carcinoma. Adjusted for age, gender, BMI, SBP, DBP, FPG, TC, TG, HDL-C, LDL-C, TgAb, TPOAb, history of exposure to ionizing radiation, and family history of thyroid cancer

Table 2 showed the adjusted association between thyroid parameters and prevalence of PTC. Higher levels of TSH, TSHI, TT4RI, TFQI, and PTFQI were significantly associated with increased risk of PTC after adjustment of age, gender, BMI, SBP, DBP, FPG, TC, TG, HDL-C, LDL-C, TgAb, TPOAb, history of exposure to ionizing radiation, and family history of thyroid cancer. With per SD increase of TSH, TSHI, TT4RI, TFQI, and PTFQI, the OR (95% CI) of PTC was 1.21 (1.13–1.29), 1.31 (1.18–1.46), 1.01 (1.01–1.02), 1.94 (1.45–2.60), and 1.82 (1.41–2.34), respectively. In contrast, higher level of FT3/FT4 ratio was significantly associated with decreased risk of PTC, and the OR (95% CI) of PTC was 0.18 (0.03–0.96) with per SD increase of FT3/FT4 ratio. There was no significant association between per SD increase of FT3, FT4 and PTC risk. Furthermore, association between quartiles of TSH, TSHI, TT4RI, TFQI, PTFQI, FT3/FT4 ratio and PTC risk was shown in Fig. 2. Given the reference quartile, OR (95% CI) of PTC in patients with highest quartile of TSH, TSHI, TT4RI, TFQI, and PTFQI reached 2.46 (1.82–3.35), 2.49 (1.83–3.39), 2.41 (1.78–3.28), 1.81 (1.33–2.46), and 1.88 (1.39–2.55), respectively. For the effects of FT3/FT4 ratio on PTC risk, however, we observed no significant association between them.

**Table 2:** Association between thyroid parameters and prevalence of PTC

Thyroid parameters	Model 1		Modeql 2	
	**OR (95% CI)**	***P* value**	**OR (95% CI)**	***P* value**
FT3 (+ 1 SD)	0.96 (0.89–1.04)	0.364	0.98 (0.90–1.06)	0.601
FT4 (+ 1 SD)	0.99 (0.97–1.02)	0.613	1.00 (0.98–1.03)	0.787
TSH (+ 1 SD)	1.22 (1.14–1.30)	< 0.001	1.21 (1.13–1.29)	< 0.001
TSHI (+ 1 SD)	1.29 (1.17–1.42)	< 0.001	1.31 (1.18–1.46)	< 0.001
TT4RI (+ 1 SD)	1.01 (1.01–1.02)	< 0.001	1.01 (1.01–1.02)	< 0.001
TFQI (+ 1 SD)	1.88 (1.42–2.49)	< 0.001	1.94 (1.45–2.60)	< 0.001
PTFQI (+ 1 SD)	1.77 (1.39–2.27)	< 0.001	1.82 (1.41–2.34)	< 0.001
FT3/FT4 (+ 1 SD)	0.27 (0.06–1.19)	0.084	0.18 (0.03–0.96)	0.045

Model 1, adjusted for age and gender; model 2, adjusted for age, gender, BMI, SBP, DBP, FPG, TC, TG, HDL-C, LDL-C, TgAb, TPOAb, history of exposure to ionizing radiation, and family history of thyroid cancer

### Association of sensitivity to thyroid hormones with the TNM staging of PTC

Among 1,457 PTC patients, 1,449 had TNM staging information, including 1,317 in stage I and 132 in stage II. Comparisons of the thyroid parameters of patients with TNM stage I and stage II tumor were summarized in Table 3. There was no statistically significant difference between the patients with stage I PTC and those with stage II PTC in terms of FT4, TSH, TSHI, TT4RI, TFQI, and PTFQI, while FT3 (*P* < 0.001) and FT3/FT4 ratio (*P* = 0.002) were significantly lower in patients with stage II PTC. Furthermore, we conducted spearman correlation analyses to investigate the correlations between FT3, FT3/FT4 ratio, and the TNM staging of PTC. The results revealed a significant negative correlation between FT3 (*r* = -0.118, *P* < 0.001), FT3/FT4 ratio (*r* = -0.081, *P* = 0.002) and the TNM staging of PTC.

**Table 3:** Comparisons of the thyroid parameters of patients with stage I and stage II PTC

Thyroid parameters	TNM staging		*P* value
	I	II	
FT3 (pmol/L)	4.95 (4.58, 5.36)	4.72 (4.25, 5.13)	< 0.001
FT4 (pmol/L)	16.79 (15.21, 18.56)	16.59 (15.10, 18.30)	0.449
TSH (mIU/L)	2.46 (1.69, 3.70)	2.54 (1.76, 3.76)	0.482
TSHI	3.19 (2.77, 3.59)	3.21 (2.86, 3.59)	0.783
TT4RI	41.18 (27.96, 62.15)	42.34 (30.10, 62.18)	0.581
TFQI	0.03 (-0.21, 0.28)	0.05 (-0.17, 0.24)	0.982
PTFQI	0.09 (-0.16, 0.38)	0.09 (-0.11, 0.32)	0.993
FT3/FT4	0.30 (0.27, 0.33)	0.29 (0.25, 0.32)	0.002

Data are expressed as median (interquartile ranges)

## Discussion

Our study demonstrated that TSHI, TT4RI, TFQI, and PTFQI was positively associated with the risk of PTC, while the association between FT3/FT4 ratio and PTC risk was negative. Additionally, FT3/FT4 ratio was found negatively correlated with the TNM staging of PTC. To the best of our knowledge, this is the first study that explore the relationship between sensitivity to thyroid hormone indices and PTC.

Previous studies yielded inconclusive results regarding the association between thyroid function and PTC risk. Our findings on the positive correlation between TSH and PTC supported the opinion that higher TSH levels led to a higher risk of thyroid cancer, while limited studies suggested such association was insignificant or negative [9, 21]. No significant association was found between FT3, FT4, and PTC in our study, consistent with previous reports [12, 22–25], whereas one report showed a markedly negative association between thyroid hormone levels and thyroid cancer risk [10]. The variability in population-based studies highlight that TSH or thyroid hormone levels alone maybe insufficient to explain the relationship between thyroid system and PTC.

Circulating thyroid hormones are controlled by hypothalamus-pituitary-thyroid (HPT) axis in a negative feedback manner. Due to the complex network regulation of the HPT axis, the calculation of composite indices can provide a more comprehensive and objective indication of thyroid hormone homeostasis than a single hormone level. Composite indices, such as TSHI and TT4RI, were initially proposed to assess the central sensitivity to thyroid hormones [20, 26]. A more stable index, TFQI, was proposed by Laclaustra et al. in 2019 [13]. To date, thyroid hormone sensitivity indices have been revealed to be associated with obesity [13, 17, 27], prediabetes [28], diabetes [13, 14], gestational diabetes [29], hypertension [14], metabolic syndrome [13], non-alcoholic fatty liver disease [30], renal function [15, 31], homocysteine levels [32], hyperuricemia [17], cardiovascular disease [17], diabetes-related mortality [13], and all-cause mortality [16].

Our study demonstrated that increased TSHI, TT4RI, TFQI, and PTFQI, indicating a decrease in central sensitivity to thyroid hormone, were significantly related to higher incidence of PTC. Notably, the correlations between TSHI, TFQI, PTFQI and PTC risk were obviously stronger than that of TSH. For example, the OR for an elevated PTC risk was 1.88 versus 1.22 per SD increase in TT4RI and TSH, respectively. Central resistance to thyroid hormone is characterized by an elevated level of circulating FT4 without TSH suppression. It is widely known that, TSH is the major regulator for thyroid function and development, which induce thyroid hormone synthesis and release and maintain trophic thyroid cell integrity [33]. In previous studies, high TSH levels were found to have a positive effect on the incidence of PTC [34], and inhibition of TSH can significantly improve the prognosis and survival time of cases with differentiated thyroid cancer [35]. In addition, both in vitro and in vivo studies have shown that thyroid hormones (T3 and T4) play important roles in tumor proliferation, invasion and angiogenesis [36].

Although the exact mechanism linking central thyroid hormone sensitivity to PTC remains unclear, there might be potential explanations as follows: since thyroid hormone resistance was associated with illnesses, e.g. obesity and diabetes [13, 14], that have a close connection with PTC [37, 38], harmful effects of metabolic diseases might serve as intermediaries to elevate the risk of PTC that induced by thyroid hormone resistance. Alternatively, central thyroid hormone resistance might be a feature of PTC rather than being a causal factor to increase PTC risk, in view of the shortcomings of a cross-sectional investigation. But unlike the acquired mild resistance to thyroid hormone, how hereditary resistance to thyroid hormone relates to thyroid cancer occurrence has been studied in earlier researches. Hereditary resistance to thyroid hormone is mainly determined by genetic variants in the thyroid hormone receptor beta (*THRB*) gene, which plays an essential role in repression of thyroid carcinoma [39]. It was reported that a transgenic mouse model harboring a dominant-negative variant of *THRB* accelerated the metastasis of thyroid carcinoma indicating the role of *THRB* in carcinogenesis. On the other side, both in vitro and in vivo experiments confirmed a delayed progression of thyroid tumors when the repressed *THRB* was rescued [40].

Furthermore, our results showed that increased FT3/FT4 ratio was correlated to a lower risk of PTC, while FT3/FT4 ratio inversely associated with the TNM staging. As the bioactive hormone, FT3 is converted from FT4 through deiodinases and has much higher affinity for thyroid receptors in the peripheral organs than T4. FT3/FT4 ratio is superior in reflecting the peripheral effect of thyroid hormones. Thus, weakened function of deiodinases, namely the resistance to thyroid hormone, might be present in patients with PTC [41]. Recent studies have shown that a low FT3/FT4 ratio indicates a shorter overall survival and progression-free survival in patients with metastatic colorectal cancer and renal cell carcinoma [42–44]. The specific mechanisms underlying the association between peripheral thyroid hormone sensitivity and PTC need to be further clarified.

There are several major limitations in our research. First of all, all subjects were recruited from a single hospital which might result in a potential selection bias. Second, this study only included patients undergoing thyroid surgery due to TNs, limiting the extrapolation of results to the general population. Nevertheless, this study is very meaningful and the results could be applied to the differential diagnosis of TNs, whose incidence is increasing in the general population. Third, to explore the real application value of sensitivity to thyroid hormone indices in general hospitalized patients with TNs, those with abnormal thyroid function were not excluded. However, we obtained consistent results in sensitivity analyses among 1,535 individuals, who were free of thyroid diseases and with FT3, FT4, and TSH levels within the euthyroid range. The multi-adjusted OR (95% CI) of PTC was 1.51 (1.22–1.85), 1.02 (1.01–1.02), 1.66 (1.16–2.37), and 1.60 (1.19–2.15) with per SD increase of TSHI, TT4RI, TFQI, and PTFQI (Supplementary Table S1). Finally, given the inherent flaws of case–control studies, the causative association between sensitivity to thyroid hormone and PTC could not be evaluated in the present study.

## Conclusion

Our study showed that decreased central thyroid hormone sensitivity was associated with a higher risk of PTC, while association between peripheral thyroid hormone sensitivity and PTC was negative. TSHI, TT4RI, TFQI, PTFQI, and FT3/FT4 ratio could be applied to predict PTC in Chinese patients with TNs. Larger sample-size cohort studies are further needed to validate our findings, and the epidemiology and mechanism of thyroid hormone sensitivity should come into light in the future.

## Supplementary Information

**Additional file 1.**

## Data Availability

The datasets used and/or analysed during the current study are available from the corresponding author on reasonable request.

## Abbreviations

PTC: Papillary thyroid carcinoma
TNs: Thyroid nodules
TSHI: Thyroid stimulating hormone index
TT4RI: TSH T4 resistance index
TFQI: Thyroid feedback quantile-based index
PTFQI: Parametric thyroid feedback quantile-based index
BMI: Body mass index
FPG: Fasting plasma glucose
TC: Total cholesterol
TG: Triglyceride
HDL-C: High-density lipoprotein cholesterol
LDL-C: Low-density lipoprotein cholesterol
FT3: Free triiodothyronine
FT4: Free thyroxine
TSH: Thyroid Stimulating hormone
TgAb: Thyroglobulin antibody
TPOAb: Thyroid peroxidase antibody
cdf: Cumulative distribution function
SBP: Systolic blood pressure
DBP: Diastolic blood pressure
HPT: Hypothalamus-pituitary-thyroid
THRB: Thyroid hormone receptor beta
