# Supplementary material for: Sensitivity to thyroid hormone indices are associated with papillary thyroid carcinoma in Chinese patients with thyroid nodules
Source: BMC Endocr Disord. 2023 Jun 1;23:126. doi: 10.1186/s12902-023-01381-8 (PMC10233979; doi:10.1186/s12902-023-01381-8)
Supplement: Supplementary file 1 — Additional file 1. [file 12902_2023_1381_MOESM1_ESM.docx]

**Supplementary Table S1** Sensitivity analysis of the association between thyroid parameters and PTC in 1,535 euthyroid subjects

| **Thyroid parameters** | **Model 1** | |  | **Model 2** | |
| --- | --- | --- | --- | --- | --- |
|  | **OR (95% CI)** | ***P* value** |  | **OR (95% CI)** | ***P* value** |
| FT3 (+1 SD) | 0.76 (0.61-0.94) | 0.013 |  | 0.75 (0.60-0.94) | 0.011 |
| FT4 (+1 SD) | 0.97 (0.92-1.03) | 0.300 |  | 0.98 (0.93-1.04) | 0.548 |
| TSH (+1 SD) | 1.32 (1.17-1.50) | <0.001 |  | 1.31 (1.15-1.49) | <0.001 |
| TSHI (+1 SD) | 1.49 (1.22-1.83) | <0.001 |  | 1.51 (1.22-1.85) | <0.001 |
| TT4RI (+1 SD) | 1.02 (1.01-1.02) | <0.001 |  | 1.02 (1.01-1.02) | <0.001 |
| TFQI (+1 SD) | 1.58 (1.12-2.23) | 0.009 |  | 1.66 (1.16-2.37) | 0.005 |
| PTFQI (+1 SD) | 1.55 (1.17-2.07) | 0.003 |  | 1.60 (1.19-2.15) | 0.002 |
| FT3/FT4 (+1 SD) | 0.22 (0.02-2.95) | 0.255 |  | 0.11 (0.01-1.64) | 0.111 |

Model 1, adjusted for age and gender; model 2, adjusted for age, gender, BMI, SBP, DBP, FPG, TC, TG, HDL-C, LDL-C, TgAb, TPOAb, history of exposure to ionizing radiation, and family history of thyroid cancer.
